# Effect of bamboo shoot dietary fiber on gel properties, microstructure and water distribution of pork meat batters

**DOI:** 10.5713/ajas.19.0215

**Published:** 2019-08-26

**Authors:** Ke Li, Jun-Ya Liu, Lei Fu, Ying-Ying Zhao, He Zhu, Yan-Yan Zhang, Hua Zhang, Yan-Hong Bai

**Affiliations:** 1 College of Food and Bioengineering, Zhengzhou University of Light Industry, Henan Collaborative Innovation Center for Food Production and Safety, Henan Key Laboratory of Cold Chain Food Quality and Safety Control, Zhengzhou, 450001, China; 2 School of Food Science and Technology, Shandong Agriculture and Engineering University, Jinan 250100, China

**Keywords:** Bamboo Shoot Dietary Fiber, Gel Property, Microstructure, Water Distribution, Meat Batter

## Abstract

**Objective:**

To develop healthier comminuted meat products to meet consumer demand, the gel properties, rheological properties, microstructure and water distribution of pork meat batters formulated with various amounts of bamboo shoot dietary fiber (BSDF) were investigated.

**Methods:**

Different levels of BSDF (0% to 4%) were added to pork batters, and the pH, color, water-holding capacity, texture and rheological properties of pork batters were determined. Then, pork batters were analyzed for their microstructure and water distribution using scanning electron microscopy (SEM) and low-field nuclear magnetic resonance (LF-NMR).

**Results:**

Compared with the control, BSDF addition into meat batters showed a significant reduction in L*-value and a significant increase in b*-value (p<0.05). BSDF addition of up to 4% reduced the pH value of pork batters by approximately 0.15 units; however, the cooking loss and expressible water loss decreased significantly (p<0.05) with the increased addition of BSDF. The hardness and gel strength were noticeably enhanced (p<0.05) as the content of BSDF increased. The rheological results showed that BSDF added into pork batters produced higher storage modulus (G′) and loss modulus (G″) values. The SEM images suggested that the addition of BSDF could promote pork batters to form a more uniform and compact microstructure. The proportion of immobilized water increased significantly (p<0.05), while the population of free water was decreased (p<0.05), indicating that BSDF improved the water-holding capability of pork batters by decreasing the fraction of free water.

**Conclusion:**

BSDF could improve the gel properties, rheological properties and water distribution of pork meat batters and decrease the proportion of free water, suggesting that BSDF has great potential as an effective binder in comminuted meat products.

## INTRODUCTION

Consumers are increasingly aware of the relationship between red meat consumption and health and highly concerned regarding the nutrition and health issues of processed meat products [1,2]. As a result, meat scientists and producers introduce bioactive components during processing and design new formulations to improve the nutritional profile of processed meat, thus improving its desirable qualities and increasing product value for the meat industry [3]. A number of different nonmeat proteins and hydrocolloids are applied to achieve healthier meat products with reduced fat, lower salt and fewer calories, as well as the inclusion of bioactive components, such as natural antioxidants and dietary fibers [1,4,5]. Meanwhile, the incorporation of various ingredients into meat products has the ability to promote meat/protein matrix formation and a high water-holding capacity (WHC) [5–7].

Dietary fiber, the “seventh nutrient”, consists of edible plant remnants or analogous carbohydrates which are difficult to digest and absorbed in the human intestinal tract; this nutrient can prevent diabetes, intestinal disorders and obesity [8]. It contains oligosaccharides, lignin, polysaccharides and associated plant substances. Dietary fiber is advantageous in improving the nutritional attributes, textural characteristics, and cooking yield of finished meat products [9], and induces sources such as wheat fiber [10], citrus fiber [11] and dried carrot pomace [12]. Additionally, when various dietary fibers are added into meat products, they reduce product cost. However, the average daily intake of dietary fibers is approximately 15 g, which is still far below the recommended value of 28 to 36 g of fiber per day for adults [9]. In recent years, many studies show that the addition of various dietary fibers into meat products improved the WHC and texture of the final products by utilizing the fiber’s functional properties, such as water retention, textural characteristics, emulsification stability, and noninfluence on flavor [13,14]. The addition of wheat fiber enhanced the gel properties of surimi [15]. However, Cardoso et al [16] reported that inner pea fiber did not improve the gel properties of mackerel surimi gels. Han et al [9] indicated that different kinds of dietary fibers, derived from various sources, have different effects on the gel properties of meat batters. Bamboo shoot dietary fiber (BSDF), a good source of functional ingredients, has been applied in milk pudding [17], fish ball [18], and frozen dough [19]. Our previous research has revealed that BSDF has a higher WHC, oil-binding capacity and swelling property compared with those of soybean fiber and rice bran fiber [19,20], which may be applied in meat products to improve both nutritional values and functional properties. Little information is available concerning the impact of BSDF on thermal gel properties and rheological properties of meat batters.

Low-field nuclear magnetic resonance (LF-NMR) transverse (T_2_) relaxometry is a direct and noninvasive technical method to detect the water mobility and distribution in a meat matrix. The spin-spin relaxation time (T_2_) is a useful metric to understand water-protein interactions in order to evaluate the factors relating to food additives and their effects on water or fat binding properties. In recent years, this metric has been applied to examine water distribution and mobility in meat batters [21]. Only a few studies have reported the water distribution of meat batters or proteins prepared with dietary fibers, such as sugarcane dietary fiber or carboxymethyl cellulose [9,22,23]. The LF-NMR analysis on the physical state of water in meat batters prepared with BSDF remains to be explored.

Therefore, this study aims to investigate the effect of BSDF on pH, colour, WHC, texture and dynamic rheological properties of pork batters, and to determine the microstructure, water distribution and mobility of pork batters to further understand the role of BSDF in the meat matrix structure and formation of the macro-quality of meat product.

## MATERIALS AND METHODS

### Raw materials and ingredients

The BSDF was obtained from Gengshengtang Ecological Agriculture Co., Ltd. (Zhejiang, China), vacuum freeze-dried for 12 h, ground to be superfine, and then collected by filtration fitted with a 100-mesh screen. The proximate compositions of BSDF were as follows: 15.15% protein, 1.83% fat, 1.35% ash, and 76.85% total dietary fiber (insoluble dietary fiber accounts for 65.78%).

Fresh pork leg lean meat (73.25% moisture, 22.62% protein, 3.20% fat, and pH 5.98) was purchased from a local market (Zhengzhou, China). All subcutaneous fat and visible connective tissues were removed. The pork meat was cut into small cubes, mixed and passed through a grinder (MM-12, Guangdong, China), affixed with a 6 mm diameter plate. The ground meat (0.5 kg each) was packaged in polyethylene bags and stored at −20°C until use within two weeks.

### Preparation of pork batters

The formulation of pork batters was made according to Table 1. The pork batters were prepared by mixing pork meat with ice/water and salt containing different amunts of BSDF. Five different pork batters (Table 1) were prepared in four replicates each on different occasions. In each replication, the ground meat (0.5 kg) within each batter formulation was thawed overnight at 0°C to 4°C and then were prepared using a Waring Blender (Grindomix GM 200, Restch, Haan, Germany). The thawed, ground meat was added to 1/3 chilled water and homogenized for 20 s at a speed of 1,500 rpm, followed by a 1 min rest. After adding salt and additional 1/3 chilled water, the batter was blended at 1,500 rpm for 20 s, followed by a 1 min rest. BSDF at different proportions (C, 0%; T1, 0.1%; T2, 0.2%; T3, 0.3%; T4, 0.4%; respectively) (Table 1) and additional 1/3 chilled water were added to the mixtures and blended for 20 s at 1,500 rpm. The procedure was then finished by blending at 3,000 rpm for 30 s (final temperature less than 10°C). Next, the prepared batter samples (~30 g) were loaded into 50 mL-capped plastic centrifuge tubes and centrifuged (Beckman Avanti J-E, Beckman Coulter, Miami, FL, USA) at 500 g for 2 min at 4°C to remove any remaining air bubbles. Half of the tubes for each meat batter formulation were heated in a water bath at 80°C for 20 min. All of the tubes containing raw or heated pork batters were subsequently chilled and stored at 4°C until testing.

### pH and color measurement

The pH of the heated pork batter samples was measured. The heated batters were cut into a 2.5-cm section. The pH value was measured using a portable pH meter (S2-Food Kit, Mettler-Toledo, Zurich, Switzerland) inserted into the geometric center of cooked batter samples. The pH values were obtained for six unique samples from each batter formulation. The color of cooked pork batters was determined according to the method of Zhuang et al [7] with slight modifications.

The color of heated pork batters was measured using a Spectrophotometer (Ci6X, X-Rite Incorporated Co., Grand Rapids, MI, USA), calibrated with a white and black calibration board. A mean of six determinations was performed for each L*-value (lightness), a*-value (redness), and b*- value (yellowness).

### Cooking loss and expressible water loss

The cooking loss (CL) of pork batters was performed according to the method of Han et al [9]. CL was measured as the weight difference before and after cooking, expressed as a percentage of the initial weight, using the following formula:

$$\text{CL}\%=\frac{\text{W}1-\text{W}2}{\text{W}1}\times 100$$

W1 and W2 are the precooking weight and postcooking weight of pork batters, respectively. Six samples were used for measurement from each batter formulation.

The expressible water loss (EWL) of cooked pork batters was determined using the compression method, as described by Carballo et al [24] with some modifications. The cooked batters were cut into 1 cm thick slices prior to being wrapped in gauze and 18 pieces of filter paper. A force of thirty-five kg was used for five min to transfer the liquids from the cooked pork batter sample to the filter papers. The EWL was determined for six replicates using the following equation:

$$\text{EWL}\%=\frac{\text{W}3-\text{W}4}{\text{W}3}\times 100$$

W3 and W4 are the weight of cooked pork batters before and after pressing, respectively. Six samples were used for measurement from each batter formulation.

### Texture profile analysis and gel strength

Texture profile analysis (TPA) was determined using a TA-XT2i Plus texture analyzer (Stable Micro System Co., Godalming, UK) equipped with a cylindrical probe (a 50 mm diameter) according to the method of Pan et al [25]. Ten heated pork batter samples (2.0 cm height and 2.5 cm diameter) per formulation were compressed twice to 50% of the original height. The instrument settings were as follows: pretest speed of 5 mm/s, test speed of 2 mm/s, posttest speed of 2 mm/s, and trigger force of 5 g. The parameters (hardness, springiness, cohesiveness, and chewiness) for TPA attributes were obtained.

The gel strength of heated pork batters was determined by a TA-XT2i Plus texture analyzer equipped with a 5.0 mm diameter aluminous cylindrical probe as described by Chen et al [26] with slight modifications. Ten heated pork batter samples (20.0 mm height) for each formulation were compressed using a trigger-type button with a 2 mm/s pretest speed, a 1.0 mm/s test speed, a 1.0 mm/s posttest speed, 10 mm distance and 5 g trigger force. The peak load was recorded after compression. The peak force corresponding to the rupture of the gel samples was defined as the gel strength (g).

### Dynamic rheological measurement

Dynamic rheological studies were performed as described by Ullah et al [27] using a rotary rheometer (DSR200, Rheometric Scientific, Newcastle, DE, USA) equipped with a 40 mm parallel plate geometry. The raw batter samples were placed between the flat parallel plates (0.5 mm gap). The samples were heated from 25°C to 80°C at 2°C/min with a programmable circulating water bath. During the heating process, the samples were continuously sheared in oscillatory mode at a fixed frequency of 0.1 Hz. Changes in storage modulus (G′) and loss modulus (G″) were monitored. All measurements were repeated three times for each batter formulation.

### Scanning electronic microscopy

The microstructure of heated pork batters was determined according to the procedure of Han et al [28]. Sections (5 mm thick) obtained from heated pork batters were fixed for 24 h in 0.1 mol/L phosphate buffer (pH 7.0) containing 2.5% glutaraldehyde at 4°C, and then dehydrated in incremental concentrations of ethanol (50%, 70%, 90%, 95%, and three times with 100%) for 10 min per solution. The microstructure of the samples was observed using a scanning electron microscope (Joel, JSM 6490LV, Tokyo, Japan) at an accelerating voltage of 20 kV. Four pictures of each batter formation were taken.

### Low-field nuclear magnetic resonance

The pork batter sample (approximately 2.0 g) was heated in a water bath (TW20, Julabo Co., Ltd., Gerhard-Juchheim-Strasse, Germany) at 80°C for 20 min and was subsequently chilled at room temperature for 12 h. Then, the sample was placed inside a 15-mm cylindrical glass tube and inserted into the NMR probe. The transverse relaxation times (T_2_) were measured on a low field pulsed analyzer (NMI20, Niumag Electric Corporation, Shanghai, China) operating at 22.6 MHz according to the method of Han et al [28]. The T_2_ measurement was performed using Carr-Purcell-Meiboom-Gill sequences at 32°C. The pulse parameters were as follows: scan repetitions were 8, echoes repetitions were 12,000, interval time between scans was 110 ms, and τ-value (between the pulse of 90° and 180°) was 250 μs. The LF-NMR T_2_ data was fitted to a multiexponential curve with the Multi-Exp Inv Analysis software (Niumag Electric Corporation, China), which uses the inverse Laplace transform algorithm. Four relaxation times (T_21_, T_22_, T_23_, and T_24_) and their corresponding water populations (P_21_, P_22_, P_23_, and P_24_) were recorded. Six samples for each batter formulation were analyzed.

### Statistical analysis

All data were presented as the mean±standard deviation. Statistical analysis of the data was analyzed by analysis of variance program using a statistical software package SPSS v.21.0 (IBM Corporation, NY, USA). The significant differences between the means of variables among different treatments were determined by Duncan’s multiple range test (p<0.05).

## RESULTS AND DISCUSSION

The pH of pork meat batters prepared with various concentrations of BSDF are presented in Table 2. The pH value of heated pork batters decreased approximately 0.15 units from 5.98 to 5.83, due to the addition of BSDF. However, previous researches found that incorporating some dietary fibers, such as cellulose nanofibers and hydrated wheat fiber, did not affect the pH of meat batter systems [29,30]. The decrease in pH was probably associated with the acidic components in BSDF. The BSDF may contain acidic compounds, such as protocatechuic acid, caffeic acid, p-hydroxybenzoic acid, and syringic acid [31].

As shown in Table 2, although the pH of meat batters reduced, the CL sharply decreased by approximately 13% when BSDF increased from 1% to 4%. According to our previous study [20], BSDF was capable of absorbing additional water (approximately 17.85 g/g), indicating that its strong water holding ability may explain the differences in CL among the treatments. Although the added water content in the control group (C) was 4% less than that in T4 (Table 1), BSDF was able to hold more water in raw meat batters and played an important role in the reduction of CL, resulting in an improved cooking yield of the final comminuted meat products. These results are similar to the findings of Zhao et al [14] and Zhuang et al [7], who reported that the increase of added dietary fibers could decrease the CL of emulsified sausages or a cooked meat model system. Carvalho et al [30] and Fang et al [13] showed that the dietary fiber, such as wheat fiber and sugarcane fiber, exhibited a higher WHC to explain the differences in CL among the treatments.

The EWL reflected the water binding capacity of cooked pork batters. The addition of BSDF to meat batters significantly decreased (p<0.05) the EWL from 45.74% to 41.87% (Table 2). BSDF concentrations greater than 3% (T3 and T4 in Table 1) efficiently reduced the EWL of cooked pork batters. Improvement in the water binding capacity of cooked batters with BSDF added was partially due to the lower added water content in T3 and T4 (Table 1). Another reason may be that the increased addition of BSDF enhanced the water-retaining capacity during cooking. Overall, BSDF has a honeycomb-like microstructure [32] to support its porosity and a large surface area capable of binding more water before cooking.

Table 2 also shows the L*-value, a*-value, and b*-value of cooked pork batters prepared with different amounts of BSDF. Increasing the amount of BSDF from 1% to 4% resulted in a smaller L*-value (p<0.05) compared with that of the control. The b*-value was significantly greater (p<0.05) when a great amount of BSDF was added. The smaller L*-value and greater b*-value were related to the color of BSDF and its content when added to meat batters. The BSDF alone (L*-value = 77.33, a*-value = 0.17, b*-value = 19.36, respectively) had a relatively smaller L*-value and greater b*-value compared to those of the control. Additionally, the smaller L*-value may be a result of the decreased CL of pork batters that occurs when BSDF is incorporated. However, no significant difference (p>0.05) in a*-value was found among all the groups, indicating that the addition of BSDF did not affect the a*-value of cooked pork batters. The observations in the color (L*-value, a*-value, and b*-value) of cooked pork batters were consistent with the findings of Huang et al [33], which reported that various amounts of rice bran added into emulsified meatballs increased the b*-value and decreased the L*-value, but did not affect the a*-value. Fang et al [13] reported that different dietary fibers induced different changes in the color of comminuted products. Zhuang et al [7] reported that the incorporation of sugarcane dietary fiber increased the b*-value of emulsified sausages but decreased the a*-value.

### Texture profile analysis and gel strength

The textural properties and gel strength of cooked meat batters with various levels of BSDF are given in Table 3. The addition of BSDF has a significant influence on hardness, springiness, and chewiness (p<0.05). The cohesiveness was not significantly affected (p<0.05) by the addition of BSDF. The hardness was not significantly different (p>0.05) among C, T1, and T2. However, the addition of either 3% (T3) or 4% (T4) BSDF in pork batter formulation increased the hardness (p<0.05) compared with that of the control. The cooked pork batters formulated with BSDF also had increased the gel strength (p<0.05) compared to that of the control (Table 3), which exhibited a similar trend as hardness. Some researchers found that the addition of rice bran fiber and sugarcane fiber improved the textural properties of meat batters [13,34]. Keenan et al [35] reported that the hardness of comminuted meat products increased as the content of inulin was increased. The increase in hardness and gel strength may be due to the ability of some dietary fibers to increase interactions between different matrix components [36]. These fibers acted as “active” fillers and binders, and thus contributed to the formation of a stronger gel network through nonspecific interactions with the primary gelling component [36]. In addition, Zhuang et al [37] found that sugarcane dietary fiber was homogenously embedded within the protein matrix and acted as an active dehydrating agent to remove water from myofibrillar proteins; then, during thermal processing, the chains of the concentrated myofibrillar proteins unfolded to promote the formation of β-sheets, leading to a stronger three-dimensional gel network. However, other researchers reported that the incorporation of dietary fibers into meat products reduced the hardness by disturbing the protein gelling fiber [23]. For example, Han found that fat-reduced meat batters with pectin or carboxymethyl cellulose had a lower hardness value than those without addtives. Schuh et al [38] also found that a high level of added carboxymethyl cellulose (3%) reduced the firmness. These dietary fibers had a weaker ability to form a strong and coherent protein network during heating, which then disrupted the protein-water and protein-protein interactions, resulting in less gel strength. Therefore, the discrepancies in hardness and gel strength among prior research may be due to the dietary fiber types, meat types, and formulations [9,13,30].

The addition of BSDF decreased the springiness from 0.82 of the control to 0.73 (T4), suggesting that the addition of up to 4% BSDF reduced the springiness of cooked pork batters. Song et al [11] showed that the springiness of low-fat meat products slightly declined with an increase in the amount of citrus fiber. The chewiness of cooked batters incorporated with BSDF incorporated at 1%, 2%, or 4% were not significantly different (p>0.05) from the control, respectively. The sample chewiness was relatively higher when 3% BSDF was added (p<0.05) compared to the other treatments (Table 3). Chewiness is a secondary parameter reflecting the hardness, which depends on other parameters [9]. This result could be because the specific ratio of BSDF, water, and pork meat in the meat matrix is capable of increasing the chewiness. The addition of various dietary fibers to comminuted meat products could change the textural properties and cause significantly different effects [9,30]. The effectiveness of different fibers in products depended on chemical structure, particle size, porosity and addition method [7,9,10]. These results (Table 3) indicated that pork batters with BSDF content greater than 2% improved the hardness and gel strength of the final product.

### Storage modulus (G′) and loss modulus (G″)

**Figure 1:**
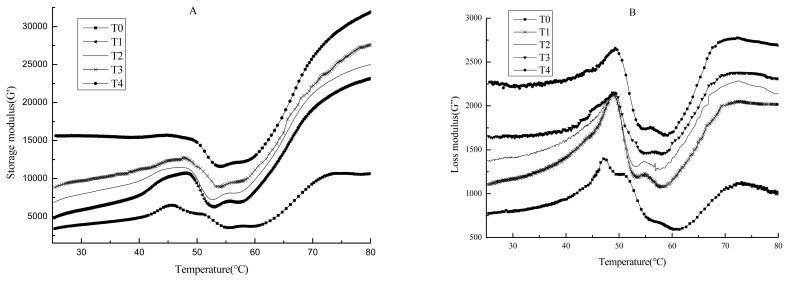
Changes in dynamic storage modulus (G′) (A) and loss modulus (G″) (B) during heating from 25°C to 80°C for pork batters with various amounts of bamboo shoot dietary fiber. BSDF, bamboo shoot dietary fiber; C, Without BSDF; T1, 1% BSDF; T2, 2% BSDF; T3, 3% BSDF; T4, 4% BSDF.

Figure 1 shows the visco-elasticity of pork meat batters with various amounts of BSDF during heating by measuring the storage modulus (G′) (Figure 1A) and loss modulus (G″) (Figure 1B). In general, the G′ of pork batters started to increase at 25°C until reaching the first peak at approximately 47°C, indicating that the preliminary and weak elastic gelation network was formed [37]. Then, the G′ decreased sharply to a minimum at approximatelyt 55°C, which was related to an increase of protein mobility and the disruption of the protein network induced by the rupture of hydrogen bonds and the unfolding of myosin tails in this temperature range [39]. The G′ increased rapidly upon further heating from approximately 55°C and plateaued at 80°C, indicating the formation of cross-links and aggregation of proteins, leading to the formation of a stable gel network [14]. The typical rheological patterns and results have been shown in previous research [40,41]. Compared with those of the control, BSDF addition (1% to 4%) resulted in greater G′ values from the initial phase to the final heat phase (Figure 1A), indicating that the use of BSDF enhanced the elasticity of composite gels. This result was due to the higher WHC of dietary fibers, leading to dehydration and interaction of partial meat proteins [40]. Additionally, BSDF contributed to the gelation network and protein matrix formation, leading to improved hardness and gel strength (Table 3).

As shown in Figure 1B, the trends of G″ were similar to those of G′. As the temperature increased from 25°C to 48°C, G″ rapidly increased and exhibited a peak. Then, G″ decreased rapidly to a minimum at 60°C. Finally, the G″ increased upon further heating to 80°C. With the increase of BSDF content, the G″ of pork batters noticeably increased as well. Choi et al [42] found that greater viscosity was correlated with the better water binding of the protein matrix. Thereby, BSDF had a great water retaining capacity which benefited to the viscosity of the gels.

### Scanning electronic microscopy

**Figure 2:**
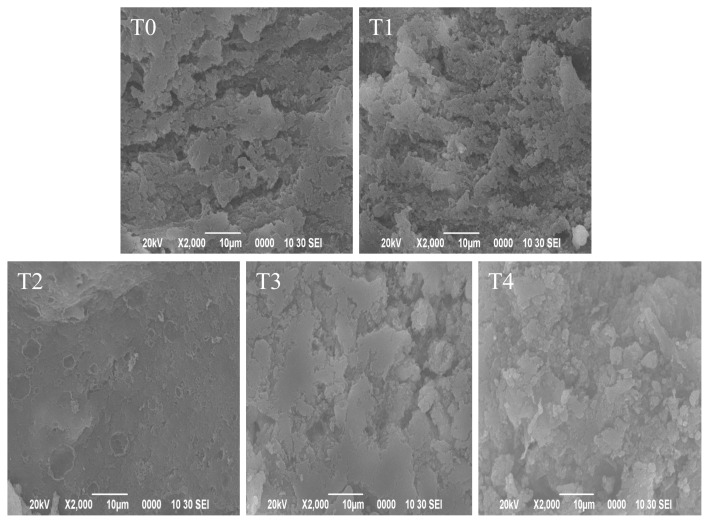
Scanning electron micrographs of heated pork batters with various amounts of bamboo shoot dietary fiber. BSDF, bamboo shoot dietary fiber; C, without BSDF; T1, 1% BSDF; T2, 2% BSDF; T3, 3% BSDF; T4, 4% BSDF.

Figure 2 shows the microstructures of cooked pork batters containing various amounts of BSDF. The micrographs indicated that different concentrations of BSDF affected the structure of pork batters. As shown in Figure 2, the control sample (C) exhibited a coarse appearance and had a large cavity network structure, which resulted in the increased water loss from the protein matrix, as previously shown [11]. Zhuang et al [22] reported that the pores in the microstructure of gels were water channels. Thereby, the large pores caused the water to easily escape from the protein network. Pork batters containing BSDF had an increased density of the gel network and gels appeared compact, uniform, and continuous compared with the control (Figure 2, T1–T4). As the concentration of BSDF increased, the cavities of water channels in the gel microstructure of gels decreased. These observations of microstructural changes were important in understanding the improvements in water-binding ability of pork batters when BSDF is added. BSDF could fill the protein matrix to retain water and enhance gel strength. Similar findings were reported by Zhao et al [43], who observed that the addition of regenerated cellulose fiber was effective in decreasing the pores in water channels and filling the protein matrix, making it easier to trap water and immobilize water. Additionally, it is necessary to note the microstructure of cooked pork batters when the concentration of BSDF reached 4% (Figure 2, T4). The network structure of gels exhibited greater density and was more compact than other treatments. It could be inferred that the added BSDF may self-aggregate within the protein matrix and have synergistic effects on the overall gel properties [8,39].

### Low-field nuclear magnetic resonance

**Figure 3:**
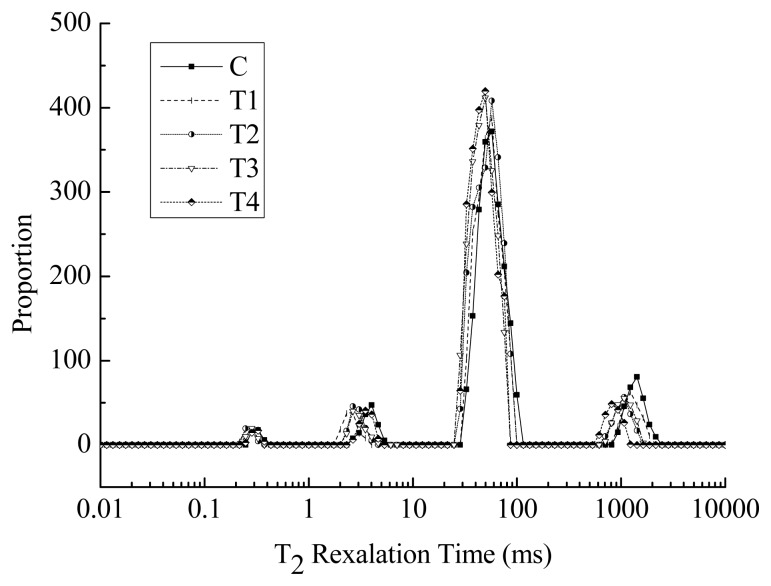
The typical distribution of nuclear magnetic resonance T_2_ relaxation times of heated pork batters with various amounts of bamboo shoot dietary fiber. BSDF, bamboo shoot dietary fiber; C, without BSDF; T1, 1% BSDF; T2, 2% BSDF; T3, 3% BSDF; T4, 4% BSDF.

Figure 3 shows distributed water proton NMR T_2_ relaxation times of cooked pork batters with various amounts of BSDF. There were four distinct water peaks in all of pork batter samples. The typical water populations centered at approximately 1 to 10 ms (T_21_ and T_22_), 40 to 70 ms (T_23_), and 300 to 2,000 ms (T_24_) were observed for the cooked meat batters [44]. According to Han et al [28], these water populations reflected the mobility of water fractions from the most tightly bound to most loosely bound, which had been assigned to the bound water (T_21_ and T_22_), immobilized water (T_23_) and free water (T_24_), respectively. The major population of water in the cooked pork batters was the immobile water (Figure 3), representing the water trapped within the protein matrix [45].

Four T_2_ relaxation times and their corresponding proportions are shown in Table 4. The addition of BSDF had no significant effect on the T_21_ relaxation time and population (P_21_) (p>0.05) in cooked pork batters. Zhuang et al [22] reported that sugarcane insoluble dietary fiber did not change the population of water tightly associated to protein (centered at 0 to 10 ms). Compared with those of the control, T_22_ relaxation times were significantly decreased (p<0.05) when BSDF was added into cooked pork batters at a 1%, 2%, or 3% concentration, while the T_22_ relaxation time did not changed (p> 0.05) in the T4 sample; these results indicated that the changes in relaxation time of T_22_ were related to the appropriate concentrations of dietary fibers. The changes in the water tightly bound to macromolecules (T_22_) corresponded to the availability of protein side-chains for water binding sites [46]. Higher level of BSDF addition (4%) may induce less availability of protein side-chains for hydrophilic interactions between BSDF and meat proteins. Higher amounts BSDF formed more self-aggregation in the meat matrix, which may explain why no effect on T_22_ was detected in the T4 sample. Compared with that of the control, the tendency of P_22_ was increased (p<0.05) with 1% to 4% BSDF was added. This may suggest that the addition of BSDF not only could improve the composite meat gel network from the shorter T_22_ relaxation time, and increase the population (P_22_) of protein-tightly associated water. There was no significant difference in T_22_ between the control and 4% BSDF (p>0.05), but P_22_ increased significantly for 4% BSDF addition to that of the control. The trends for T_22_ and P_22_ were not consistent between 4% BSDF and the control. Previous studies have also shown that specific alterations or trends were not always consistent between the relaxation times and their corresponding proportions [22,46]. The P_22_ value was improved due to the capacity of 4% BSDF to bind more water because of the increased number of hydrophilic groups, although higher amounts of BSDF easily formed self-aggregation in the meat matrix. Zhuang et al [37] found that as the concentration of 40-mesh sugarcane dietary fiber increased, the proportion of immobilized water in gels was enhanced, which was attributed to the fibers possessing polar groups that bind water. In the cooked pork batters, the amount of BSDF added contributed to significant differences in T_23_, T_24_, P_23_, and P_24_ (p<0.05). Compared with those of the control, T_23_ and T_24_ relaxation times were significantly decreased (p< 0.05) as the BSDF concentration increased from 1% to 4%. The proportion of P_23_ was enhanced from 79.81% to 86.91% (p<0.05), while P_24_ was reduced from (p<0.05) 13.95% to 6.37%. This finding was consistent with the report of Song et al [11] which utilized citrus fiber to improve the water-binding ability of low-fat frankfurters. The shorter T_21_ and T_22_ relaxation times indicate that BSDF added into pork batters could improve the binding capacity of water with macromolecules, and the increased proportion of immobilized water and reduced proportion of free wate contribute to an increased cooking yield from the meat gels. Overall, LF-NMR and scanning electronic microscopy (SEM) analyses suggested that BSDF prevents the water exudation by holding and trapping water within the meat protein matrix, and decreases the number of water channels by promoting a compact gel network. Therefore, the addition of BSDF improves the WHC of cooked pork batters.

## CONCLUSION

The gel properties, WHC, dynamic rheological behaviors, microstructure, water mobility, and water retention of pork meat batters were significantly affected by the addition of BSDF. The addition of BSDF significantly reduced CL and EWL of pork batters, improved its texture and increased G′ and G″. The SEM analysis revealed that the addition of BSDF contributed to a more uniform and compact microstructure of pork batters. LF-NMR analysis showed that the T_23_ and T_24_ decreased with the addition of BSDF, indicating that pork batters formulated with BSDF could bind water more firmly. Additionally, the free water fraction of meat batters was decreased by the addition of BSDF. These results suggested that the addition of BSDF effectively improve the gel properties and WHC of pork batters and can be used in comminuted meat products.

## Figures and Tables

**Table 1:** Formulations of pork batters prepared with various amounts of bamboo shoot dietary fiber

Ingredient (g)	C1)	T1	T2	T3	T4
Pork leg meat	65	65	65	65	65
NaCl	2.5	2.5	2.5	2.5	2.5
Ice water	32.5	31.5	30.5	29.5	28.5
BSDF	0	1	2	3	4
Total	100	100	100	100	100

BSDF: bamboo shoot dietary fiber.

1) C, without BSDF; T1, 1% BSDF; T2, 2% BSDF; T3, 3% BSDF; T4, 4% BSDF.

**Table 2:** pH, water-holding capacity and color parameters of heated pork meat batters with various amounts of bamboo shoot dietary fiber

Sample1)	pH	CL (%)	EWL (%)	L^*^-value	a^*^-value	b^*^-value
C	5.98±0.01^a^	26.26±0.98^a^	45.74±1.02^a^	78.96±0.39^a^	1.90±0.14	12.21±0.11^e^
T1	5.93±0.02^b^	23.38±0.69^b^	44.46±1.54^a^	77.95±0.22^b^	1.92±0.08	13.95±0.13^d^
T2	5.92±0.02^b^	19.39±0.93^c^	44.41±0.24^a^	77.44±0.60^b^	1.93±0.02	15.14±0.13^c^
T3	5.87±0.01^c^	15.95±0.90^d^	41.81±0.59^b^	76.23±0.56^c^	1.81±0.10	16.52±0.03^b^
T4	5.83±0.01^d^	13.55±0.60^e^	41.87±0.88^b^	76.36±0.62^c^	1.90±0.07	17.16±0.01^a^

BSDF, bamboo shoot dietary fiber; CL, cooking loss; EWL, expressible water loss.

1) C, without BSDF; T1, 1% BSDF; T2, 2% BSDF; T3, 3% BSDF; T4, 4% BSDF.

a–e Different letters indicate significant differences among the means in the same column (p<0.05).

**Table 3:** Texture profile analysis and gel strength of cooked pork batters with various amounts of bamboo shoot dietary fiber

Sample1)	Hardness (g)	Springiness	Cohesiveness	Chewiness	Gel strength (g)
C	3,828.83±134.74^b^	0.82±0.01^a^	0.52±0.02	1,640.12±70.45^bc^	1,687.32±84.91^c^
T1	3,879.68±169.37^b^	0.80±0.02^ab^	0.51±0.03	1,581.07±61.33^c^	2,003.52±121.33^b^
T2	4,004.10±51.11^b^	0.79±0.02^b^	0.52±0.02	1,689.35±40.36^b^	2,299.17±47.72^a^
T3	4,271.76±113.80^a^	0.79±0.01^b^	0.53±0.02	1,808.58±69.14^a^	2,396.31±190.98^a^
T4	4,361.58±102.13^a^	0.73±0.01^c^	0.50±0.03	1,641.10±25.12^bc^	2,425.65±97.02^a^

BSDF, bamboo shoot dietary fiber.

1) C, without BSDF; T1, 1% BSDF; T2, 2% BSDF; T3, 3% BSDF; T4, 4% BSDF.

a–c Different letters indicate significant differences among the means in the same column (p<0.05).

**Table 4:** Nuclear magnetic resonance transverse relaxation times and corresponding peak area fractions of cooked pork meat batters formulated with various amounts of bamboo shoot dietary fiber

Sample1)	T_21_ (ms)	T_22_ (ms)	T_23_ (ms)	T_24_ (ms)	P_21_	P_22_	P_23_	P_24_
C	0.31±0.12	3.77±0.30^a^	57.22±2.70^a^	1,371.32±92.31^a^	1.42±1.28	4.82±0.04^b^	79.81±1.46^d^	13.95±0.76^a^
T1	0.24±0.10	2.54±0.16^b^	55.98±3.04^a^	1,232.85±68.22^b^	1.76±1.84	4.87±0.05^b^	83.85±0.61^c^	9.52±0.17^b^
T2	0.25±0.08	2.85±0.23^b^	52.25±3.85^a^	1,152.56±92.71^b^	1.89±1.71	5.27±0.08^a^	85.02±0.47^b^	7.82±0.45^c^
T3	0.27±0.11	2.85±0.23^b^	46.53±3.55^b^	1,037.35±69.83^c^	1.84±1.06	5.14±0.18^a^	85.74±2.54^ab^	7.27±0.53^c^
T4	0.31±0.06	3.84±0.31^a^	44.91±3.24^b^	811.13±76.46^d^	1.59±1.43	5.13±0.11^a^	86.91±1.15^a^	6.37±0.60^d^

BSDF, bamboo shoot dietary fiber.

1) C, Without BSDF; T1, 1% BSDF; T2, 2% BSDF; T3, 3% BSDF; T4, 4% BSDF.

a–d Different letters indicate significant differences among the means in the same column (p<0.05).
